# Predictors of In Vitro Fertilization Outcomes in Women with Highest Follicle-Stimulating Hormone Levels ≥12 IU/L: A Prospective Cohort Study

**DOI:** 10.1371/journal.pone.0124789

**Published:** 2015-04-13

**Authors:** Lina N. Huang, Sunny H. Jun, Nathalie Drubach, Michael H. Dahan

**Affiliations:** 1 Department of Obstetrics and Gynecology, McGill University, Montreal, Quebec, Canada; 2 Department of Obstetrics and Gynecology, Stanford University, Stanford, California, United States of America; University of Kansas Medical Center, UNITED STATES

## Abstract

**Objective:**

The purpose of this study is to evaluate factors predictive of outcomes in women with highest follicle-stimulating hormone (FSH) levels ≥12 IU/L on basal testing, undergoing in vitro fertilization (IVF).

**Methods:**

A prospective cohort study was conducted at Stanford University Hospital in the Reproductive Endocrinology and Infertility Center for 12 months. Women age 21 to 43 undergoing IVF with highest FSH levels on baseline testing were included. Donor/Recipient and frozen embryo cycles were excluded from this study. Prognostic factors evaluated in association with clinical pregnancy rates were type of infertility diagnosis and IVF stimulation parameters.

**Results:**

The current study found that factors associated with clinical pregnancy were: increased number of mature follicles on the day of triggering, number of oocytes retrieved, number of Metaphase II oocytes if intracytoplasmic sperm injection was done, and number of embryos developed 24 hours after retrieval.

**Conclusions:**

Our findings suggest that it would be beneficial for women with increased FSH levels to attempt a cycle of IVF. Results of ovarian stimulation, especially embryo quantity appear to be the best predictors of IVF outcomes and those can only be obtained from a cycle of IVF. Therefore, increased basal FSH levels should not discourage women from attempting a cycle of IVF.

## Introduction

A woman’s ovarian reserve refers to her reproductive capacity as a function of the number and quality of her oocytes [1]. Each woman has a definitive amount of oocytes at birth and they gradually decrease throughout her reproductive lifetime, a process known as reproductive aging [2]. Female fertility decreases with age, although it is difficult to determine the pace at which each woman's individual number of oocytes declines [3]. Women who fail to respond adequately to standard fertility treatments, thus making it difficult to collect adequate follicles, are called "poor ovarian responders". While aging is associated with diminishing ovarian reserve leading to a natural decline in fertility, it is not always the sole contributing factor. This loss of oocytes may occur in younger women and it may happen spontaneously. The etiology of diminished ovarian reserve in women remains unclear. Previous studies have shown that a decreased amount of oocytes can be preceded by pelvic radiation, chemotherapy, and surgery [4]. Decreased ovarian reserve was also found to have an association with genetic abnormalities such as an abnormality of the X chromosome or a FMR1 premutation [4,5].

A review of the literature showed that the prevalence of poor ovarian responders in women with infertility ranges between 9 and 24% [6]. Women with diminished ovarian reserve who attempt to become pregnant with in vitro fertilization (IVF) are known to have poorer outcomes. They have been found to have higher levels of basal follicle-stimulating hormone (FSH), and to respond poorly to ovarian stimulation [7]. They have lower pregnancy rates than women in the same age range with normal ovarian reserve [8]. Women with lower oocyte counts also have much higher rates of pregnancy loss compared to age-matched women with normal ovarian reserve [9]. Women with increased FSH also have a higher risk of fetal aneuploidy, which suggests that these women not only have lower quantities of oocytes as a result of follicle attrition, but also have oocytes of lower quality compared to women with normal ovarian reserve [10].

Day 2 to 5 basal serum FSH level is used as a predictor of ovarian reserve. Women who present to infertility clinics are commonly screened for baseline FSH levels among other tests. Due to a growing trend of women delaying childbearing, an increasing number of women who present to fertility clinics have elevated levels of FSH, suggesting diminished ovarian reserve. Increasing age is known to be a negative prognostic factor for successful pregnancy [11]. Diminished ovarian reserve, independent of age, is also known to decrease pregnancy rates [8,12]. It is highly important to better understand the constellation of factors that affect the chances of women with decreased ovarian reserve to achieve pregnancy with IVF.

Prognosis of outcomes solely based on women’s age and increased FSH levels can lead to risks of multiple pregnancies or futile treatment. Couples may also have false expectations of success or, inversely, underestimate their individual chances of achieving pregnancy. Despite many studies that look at predictors of IVF outcomes, there are currently no published papers analyzing predictors of pregnancy rates in women with diminished ovarian reserve manifested by increased FSH. However, if diminished ovarian reserve is the cause of up to 50% of cancelled IVF cycles, then it would be highly important to further evaluate predictors of successful pregnancy outcomes in this population [13].

Predictors of IVF outcomes have been studied in women with normal ovarian reserve. Patient age and embryo parameters have previously been associated with IVF outcomes [11,14]. Banerjee et al. evaluated predictors of IVF outcomes in couples and found that the highest prognostic variables for successful pregnancy were age of patient, total motile sperm count, body mass index, day-3 serum FSH levels and antral follicle count [15]. Another study evaluated IVF stimulation parameters and found more than 30 predictors of live births [16]. Among these predictors, rate of blastocyst development, total amount of gonadotropins administered and number of eight-cell embryos were most correlated with live births. As expected, diminished ovarian reserve was negatively associated with live births [16].

Evaluating factors that can influence IVF outcomes in women with increased FSH will potentially aid in counselling and possibly modify their treatment in order to optimize their chances of achieving a live birth. Without further information on prognostic factors, some women with diminished ovarian reserve may not realize that their personal success rates may be higher than those based solely on their age and FSH level [15]. On the other hand, certain women may have high false hopes of achieving pregnancy. Therefore, it is important to obtain additional information on the factors that affect prognostic outcomes in order to be able to adequately counsel women between the choices of IVF treatment, donor-oocytes or adoption. The goal of this study is to determine factors that predict IVF pregnancy rates in women with increased FSH.

## Material and Methods

### Patient selection criteria

At the Stanford Reproductive Center, basal FSH levels of 12 IU/L were previously noted to be associated with lower clinical pregnancy rates. This was the cut-off used to select the women with increased FSH enrolled in this study. The study was approved by the Stanford University Committee for the Protection of Human Research Subjects. Patients signed consents approved by the IRB to participate

A prospective cohort study was conducted at Stanford University Hospital in the Reproductive Endocrinology and Infertility Center for 12 months. Women age 21 to 43 with maximum day 2 to 5 serum FSH levels who underwent IVF were enrolled. The highest serum day 2 to 5 FSH level determined eligibility. During the time period of the study, eight hundred and sixty-six patients underwent fresh IVF cycles. Donor/Recipient and frozen embryo cycles were excluded from this study. All patients at the center underwent day 2 to 5 serum FSH screening. These levels were usually assessed at their initial infertility evaluation and were obtained within six months of treatment. Serum Anti-Müllerian Hormone levels were not performed because in all cases these women clearly had evidence of decreased ovarian reserve. The assay is only performed in women whose baseline follicle counts are above 7 or serum baseline FSH levels are below 12 IU/L. Since the goal of this study was to address the exclusive role of basal serum FSH, the data collection was blinded to antral follicle counts. Therefore, this data was not collected at the request of the IRB.

### Stimulation protocols

The controlled ovarian hyperstimulation protocol consisted of pre-treatment with oral contraceptive pills with overlapping gonadotropin-releasing hormone (GnRH) agonist down-regulation followed by FSH/hMG, microdose flare agonist, or antagonist protocols. None of the patients received a minimal or mild stimulation protocol. All patients received injections of recombinant human growth hormone, 8 units, daily, (Merck emd, Massachusetts) starting 15 days before stimulation. Given that these patients were expected to be poor responders and had been counselled on the low chance of success, no cycles were cancelled for minimal results from stimulation. A greatly improved follicular response in future cycles was not anticipated. Oocytes were inseminated conventionally or by intracytoplasmic sperm injection (ICSI) if performed, 3 to 4 hours after oocyte retrieval. Embryos were cultured under mineral oil in groups, in 150 μL droplets of P1 medium (Irvine Scientific, Santa Anna, CA, USA) or Quinn’s Advantage Cleavage medium (Cooper Surgical, Trumbull, CT, USA) with 10% Serum Substitute Supplement (SSS) or 10% Serum Protein Substitute (SPS). Embryos were maintained at 37°C in a 5% O2, 5% CO2 and 90% N2 environment for 72 hours. For the blastocyst transfer group, the embryos were transferred on day 3 to Blastocyst medium (Irvine Scientific) or Quinn’s Advantage Blastocyst medium (Cooper Surgical) with 10% SSS or 10% SPS and cultured for 48 hours before transfer.

### IVF parameters

Embryo transfer was performed using a Tefcat catheter (Cook IVF, Spencer, IN, USA). Three physicians performed the transfers, each used a similar technique. The catheter was used to deposit the embryos 1.5 to 2 cm from the fundus under transabdominal ultrasound guidance. The transfer volume was 20–30μL. Clinical pregnancies were defined by seeing a gestational sac on transvaginal ultrasound at 6–7 weeks gestational age.

Several patient parameters were analyzed for their association with clinical pregnancy including age, gravity, term deliveries, maximum day 2 to 5 serum FSH levels and accessory infertility diagnoses. In addition, cycle characteristics including stimulation protocol, total gonadotropin dose, duration of stimulation in days, endometrial thickness on day of hCG injection, number of mature follicles, number of oocytes retrieved, number of mature (metaphase II) oocytes, fertilization rate and number of embryos transferred.

Serum baseline FSH values were determined by using a solid phase two-site chemiluminescent immunometric assay, run on the Immulite 2500 (Siemens Healthcare Diagnostics, Inc.). The range for testing is up to 170 mlU/ml and the sensitivity is 0.1 mlU/ml. Th Immulite 2500 usesa solid-phase, two-site chemiluminescent immunometric assay (sensitivity 0.1 IU/L, intra and inter-assay coefficients of variation of 4.2% and 7.9%, respectively).

### Statistical analysis

All statistical analyses were performed with the Statistical Package for Social Sciences 11.0 (SPSS Inc., Chicago, IL). Continuous variables were evaluated for normal distribution using the Kolmogorov-Smirnov test. Results are reported as mean value ± standard deviation (SD). Student’s t-test was used for comparison of nominal data. Levene’s test for equality of variances was applied to the data and the corresponding t-test and p values were accepted depending on whether the variances were equal. The Chi-squared with or without Fishers’s correction was used depending on cell sizes greater or less than 5 to compare non-continuous variables. Stepwise logistic regression was used to determine predictors of clinical pregnancy and miscarriage while controlling for the effect of the other variables analyzed and multiplicity. Statistical significance was accepted as a two-sided p ≤ 0.05.

This study was approved by the Stanford IRB number 6178. All patients signed the approved consent for participation, as agreed to by the IRB.

## Results

All continuous data were normally distributed by the Kolmogorov—Smirnov test.

### Demographics

Fifty-eight individuals with highest day 2 to 5 serum FSH levels ≥12 IU/L underwent eighty-two IVF cycles during the duration of the study. No individuals included in the study underwent more than 4 IVF cycles. Seventeen individuals went through two IVF cycles, two individuals underwent three IVF cycles and one individual had four IVF cycles. The study consisted of forty regular IVF cycles and forty-two ICSI cycles. The pregnancy rate was 15.9% per cycle and 22.4% per patient. The clinical pregnancy rate was 9.8% per cycle and 14.0% per patient. 3.7% of cycles resulted in miscarriage, which had a rate of 22% per pregnancy. 2.4% of cycles had ectopic pregnancies for a total rate of 15% per pregnancy. Table 1 compares the demographic factors of subjects who did or did not achieve a clinical pregnancy. No parameter differed significantly between the two groups. It is important to highlight that the mean highest basal serum FSH levels in those who did and did not conceive were almost exactly the same, 16.0 vs. 16.4 mIU/ ml respectively.

**Table 1:** A comparison of baseline characteristics from individuals with and without a clinical pregnancy, whose highest serum FSH levels was ≥12 IU/L.

	Clinical Pregnancy	Not Pregnant	p =
**Age (years)**	36.9 ± 4.8	38.6 ± 3.7	0.23
**Gravidity**	0.5 ± 0.93	1.0 ± 1.0	0.23
**Full Term Deliveries**	0.1 ± 0.4	0.4 ± 0.6	0.27
**Highest FSH (IU/L)**	16.0 ± 3.1	16.4 ± 4.2	0.78

### Infertility Diagnosis

In addition to decreased ovarian reserve, 11% of individuals presented with a diagnosis of endometriosis. Another 6% of them presented with tubal factor infertility and 27% had male factor infertility. None of the patients had recurrent pregnancy loss. Patients were more likely to have a clinical pregnancy if they had male factor infertility than if they did not (22% vs. 5%, p = 0.03). However, clinical pregnancy rates were not increased in women with tubal factor infertility (0% vs. 10%, p = 1.0), or endometriosis (0% vs. 12%, p = 0.59) compared to individuals without these diagnoses.

### IVF Stimulation Parameters

GnRh-antagonist protocols (Merck, New Jersey, or Merck emd, Massachusetts) constituted 57% of cycles, GnRh- agonist microdose flare protocols (Leuprolide acetate, Abbvie INC, Chicago, IL) made up 40% of cycles and the remaining 3% of cycles were long GnRh-agonist down regulation protocols (Leuprolide acetate, Abbvie INC, Chicago, IL). Patients were not more likely to have a clinical pregnancy if they received a GnRh-antagonist cycle (p = 1.0), or a micro-dose GnRh agonist flare protocol (p = 0.47), when compared to the other two protocols. IVF stimulation parameters comparing individuals with and without a clinical pregnancy are presented in Table 2. IVF parameters that were increased in subjects with clinical pregnancy rates were: number of mature follicles on the day of triggering, number of oocytes retrieved, number of MIIs if ICSI was done, and number of embryos which developed 24 hours after retrieval. Treatment with ICSI did not improve the clinical pregnancy rates compared to standard IVF (12% vs. 8% respectively, p = 0.71). Assisted hatching techniques did not alter the likelihood of clinical pregnancy when compared to individuals whose embryos were not assisted in hatching (9% vs. 17% respectively, p = 0.47). Only 3.6% of individuals included in the study were able to have a blastocyst transfer.

**Table 2:** Results of the statistical model to evaluate risk factors for clinical pregnancy and no pregnancy in women whose highest serum FSH levels was ≥12 IU/L.

	Clinical Pregnancy	Not Pregnant	p =
**Gonadotropin dose IU/cycle**	5933 ± 1527	7047 ± 1645	0.14
**Mean number of IVF cycles**	1.8 ± 1.5	2.3 ± 1.8	0.48
**Number of days of stimulation**	11.4 ± 1.7	12.5 ± 3.4	0.38
**Endometrial thickness at last ultrasound prior to retrieval (mm)**	10 ± 2.6	9 ± 2.0	0.15
**Number of follicle ≥15mm diameter on day of hCG**	8.0 ± 4.5	5.7 ± 2.9	0.05*
**Number of oocytes retrieved**	7.3 ± 7.3	4.1 ± 2.8	0.005 *
**Number of MIIs**	6.4 ± 3.6	3.3 ± 2.7	0.029 *
**Percent fertilization**	68 ± 23	58 ± 31	0.411
**Number of embryos**	5.0 ± 4.2	2.4 ± 2.2	0.006 *
**Number of embryos transferred**	2.6 ± 1.8	2.0 ± 1.4	0.25

* denotes statistical significance

### Prediction of clinical pregnancy and miscarriage

Stepwise logistic regression was performed to determine if any continuous variable predicted likelihood of clinical pregnancy or miscarriage while controlling for other variables and multiplicity. This was performed for all IVF cycles and then only the subjects from the first IVF cycle in order determine whether subjects with multiple cycles were exerting undue influence on the data distribution and interpretation. The results are shown in Table 3. It should be noted that number of mature follicles on the day of triggering, number of oocytes retrieved, number of MIIs if ICSI was done and number of embryos which developed 24 hours after retrieval were significant predictors of clinical pregnancy in the analysis of all IVF cycles. Results were similar in the analysis of the first IVF cycle. However, it should be noted that two additional predictors demonstrated themselves with this analysis, highest basal serum FSH level and number of embryos transferred. Only the number of full term deliveries and the highest serum level of FSH discriminated between women who had a miscarriage and those who did not, in the analysis of all IVF cycles. The number of full-term deliveries in the group who experienced a miscarriage when compared to those who did not was 1.7 ± 0.3 vs. 0.5 ± 0.3, respectively. The serum FSH level was higher in women who miscarried (20.3 ± 3.0 IU/L) compared to those who did not miscarry (15.5 ± 0.9 IU/L). In the analysis of the first IVF cycle, results remained similar with the exception of the days of stimulation becoming a significant predictor of miscarriage, with more days being a negative prognostic factor (12.5 ± 3.5 vs. 8.5 ± 0.7 days) (no miscarriage vs. miscarriage). It should also be noted that the importance of previous pregnancies went from marginal significance (p = 0.06) to significant (p = 0.04) in this analysis as well.

**Table 3:** Results of the statistical model to evaluate risk factors for clinical pregnancy and miscarriage in women whose highest serum FSH levels was ≥12 IU/L.

	Clinical Pregnancy All Cycles	Clinical Pregnancy First IVF Cycle^a^	Miscarriage All Cycles	Miscarriage First IVF Cycle^a^
**Age (years)**	0.40	0.57	0.78	0.78
**Gravidity**	0.23	0.26	0.06	0.04*
**Full term deliveries**	0.50	0.71	0.03 *	0.02*
**IVF cycle number**	0.65	__	0.65	__
**Highest FSH (IU/L)**	0.16	0.05*	0.05 *	0.06
**Gonadotropin dose per cycle**	0.20	0.21	0.72	0.10
**Days of stimulation**	0.41	0.39	0.20	0.03*
**Endometrial thickness**	0.61	0.57	0.45	0.41
**Number of follicle ≥15mm diameter on day of hCG**	0.03 *	0.06	0.24	0.66
**Number oocytes retrieved**	0.004 *	0.01*	0.28	0.72
**Number of MIIs**	0.03 *	0.05*	0.71	0.48
**% fertilization**	0.40	0.58	0.15	0.59
**Number of embryos**	0.003 *	0.01*	0.21	0.98
**Number of embryos transferred**	0.44	0.04*	0.46	0.60

*denotes statistical significance

^**a**^ For first IVF cycle analysis, the data point IVF cycle number is a constant and was not included in the analysis.

An analysis of the embryo quality was also performed. There were not enough blastocyst transfers to perform analysis on this group. However, in the cleavage stage transfers (day 3), when evaluating the number of embryos obtained: the number of 8 cell embryos (p = 0.290, p = 0.50), the number of 6 to 9 cell embryos (p = 0.44, p = 0.68), number of 10 cell embryos (p = 0.16, p = 0.54), number of embryos of 5 or less cells (p = 0.83, p = 0.25), did not differ for the clinical pregnancy or miscarriage groups respectively. To be noted, all cleavage transfers were performed day 3. Evaluation of the number of cells and the grade of the best embryo transferred (p = 0.74, p = 0,74), the second best embryo transferred (p = 0.33, p = 0.62), the third best embryo transferred (p = 0.86, p = 0.98) and the fourth best embryo transferred (P = 0.15, P = 1.0), did not differ between the clinical pregnancy and miscarriage groups. Not all subjects had 3 or even 4 embryos transferred. The analysis of these numbers was performed in subjects which did have these number transferred.

### Determination of FSH limit for decreased ovarian reserve

Serum day 2 to 5 FSH results are a continuum, with an expected resultant decreased ovarian response with increasing FSH levels. However, decreased ovarian reserve is generally defined as an FSH level cut-off at which 5% of women conceive a pregnancy. The population included in this study had a clinical pregnancy rate of 9.8% and therefore represent a group with somewhat intermediate ovarian reserve. However, it should be noted that the basal FSH cut off was correctly applied in this population, since pregnancy rates were low, in this relatively young group. Table 4 shows the likelihood of pregnancy and clinical pregnancy in women with differing maximum serum FSH level. It is not until a maximum serum FSH level of ≥ 18 IU/L is reached that decreased ovarian reserve occurs by the definition of a clinical pregnancy rate of less than 5%. It should be noted that this probability as a function of highest FSH is not linear since, as women who did not conceive at lower FSH levels drop out of analysis, probability of pregnancy may increase.

**Table 4:** Pregnancy and clinical pregnancy rates in women with maximum serum FSH level at a specific cut off or higher. ^a^

Maximum FSH (IU/L)	≥12	≥13	≥14	≥15	≥16	≥17	≥18
**Pregnancy percentage**	15.9%	17.4%	20.4%	17.6%	16.2%	13.3%	13.0%
**Clinical pregnancy percentage**	9.8%	11.6%	13.0%	11.8%	9.1%	6.7%	4.3%

^a^Miscarriage rates increased as maximum serum FSH levels rose to the level where clinical pregnancies were less than 5%

## Discussion

Up to 50% of cancelled IVF occur due to decreased ovarian reserve [13]. Therefore, for patient counselling it is important to evaluate what variables influence the chances of pregnancy in women with this criteria for being diagnosed as a poor responder. The dearth of literature looking at predictors of IVF outcomes in women with increased FSH further re-enforces the need to evaluate this group of women. Therefore, the aim of this study was to determine the factors correlated with clinical pregnancy in women with diminished ovarian reserve, manifested by high FSH levels.

In the current study, it was found that factors associated with clinical pregnancy were: increased number of mature follicles on the day of triggering, number of oocytes retrieved, number of MIIs if ICSI was done, and number of embryos developed 24 hours after retrieval. These results suggest that clinical pregnancy is influenced not by parameters available pre-stimulation. It is the response to stimulation itself which predict whether the patient will conceive a clinical pregnancy. In a study by Thum et al., women with elevated serum FSH levels had lower numbers of oocytes retrieved [17]. They were also shown to have lower clinical pregnancy and live birth rates compared to women with FSH levels in the normal range. However, women with an elevated basal FSH level who responded well to gonadotropin stimulation and generated a good number of oocytes/embryos had similar chances of becoming pregnant and having a live birth as women in the same age range with normal levels of FSH [17]. The investigators concluded that women should not be denied an IVF trial based solely on their FSH levels, especially since a portion of them may respond well to the treatment and be able to give birth to a live baby. The findings in this study also suggest that patients should be treated irrelevant of maximum serum FSH levels since it is the parameters related to the response to stimulation which will predict success. Although, it should be noted that pregnancy rates were on the lower side compared to average results at Stanford, during the time period.

Many investigators have studied predictors of IVF in infertile women, regardless of their ovarian reserve. A systematic review by van Loendersloot et al. found that female age, duration of subfertility, basal FSH levels and number of oocytes, which are all factors that reflect ovarian function, were the best predictors of IVF success [11]. Their results suggested that, higher quality embryos were also associated with higher pregnancy chances [11]. Another group of investigators, Cai et al. analyzed independent predictors of IVF outcomes in a multivariable model [14]. Nine variables were included in their final model: total number of good-quality embryos, total number of embryos, age, antral follicle count, fertilization rate, duration of infertility, endometrial thickness, number of 10 to 14-mm follicles and progesterone level on the day of hCG injection [14]. Their model showed that the quality and quantity of embryos were the two most important predictors of the cumulative outcome in IVF/ICSI and that their influence increased with advancing female age [14]. Banerjee et al. evaluated couples' predictors of outcome prior to IVF treatment and found that variables with the highest prognostic contributions were patient age, total motile sperm count, body mass index, day 3 serum FSH levels and antral follicle count [15]. Another study evaluated IVF stimulation parameters and found more than 30 predictors of live births [16]. Among the predictors, rate of blastocyst development, total amount of gonadotropins administered and number of eight-cell embryos were best associated with live births [16]. As expected, diminished ovarian reserve correlated negatively with live births [16]. Both Banerjee and Choi suggest that each couple presenting for infertility treatments should receive their own personalized predictor model. They concluded such models would allow patients to avoid futile treatment or avoid underestimating a couple's individual chances of success in achieving a live birth.

After reviewing the published predictors of IVF outcomes in infertile women, the quantity of oocytes and embryos appear to be recurrent variables influencing clinical pregnancy rates [11,14,16]. Given similar results were noted in this study involving women with decreased ovarian reserve, it seems important to explore in greater depth the influence of oocytes and embryos on pregnancy rates. A systematic review by Rienzi et al. further analyzed the predictive value of oocyte morphology [18]. They looked at fifty articles examining the effect of human oocytes on assisted reproductive technology (ART) and did not find any predictive value of non-invasive morphological features of MII phase oocytes for selecting developmentally competent oocytes [18]. Their review suggests that the quality of embryos have a higher predictive value on clinical pregnancy rates than oocyte numbers. These findings regarding embryo quality likely apply to women with increased FSH levels. This also reinforces the concept that these women should be given a chance at an IVF cycle.

Among the remaining factors evaluated in the current study, no association was found between type of ovarian stimulation and clinical pregnancy. Given that the population studied had increased FSH and are likely to be poor responders, the regimen itself may not significantly lead to alterations in the number of follicles stimulated, since all would have been treated with maximum effective gonadotropin doses. Therefore, factors influencing clinical pregnancy rates in women with increased FSH likely lies in other factors than their stimulation regimens. There was also no association between type of infertility and clinical pregnancy, with one exception. Couples with male factor infertility were more likely to conceive a clinical pregnancy. This is likely due to the fact that intracytoplasmic sperm injection is often needed to overcome male factor. Therefore couples with this diagnosis were prevented from conceiving spontaneously. Thus, this finding represents a selection bias where the couples with the best prognosis could not conceive without treatment. It was interesting that this finding did not repeat in women with tubal factor infertility. It can be hypothesized that in women with high serum FSH levels, the cause of tubal factor may also be affecting ovarian function further limiting responses to stimulation, as for example with endometriosis. A review of studies on primary or secondary infertility showed no statistically significant associations with clinical pregnancy rates after IVF [11]. The same review attempted to summarize findings for indications for IVF (tubal, male, endometriosis) and were unable to calculate odd ratios because each studies used different reference categories.

It should be noted that female age was not found to be a significant predictor of clinical pregnancy or miscarriage in this study while it has been a prognostic factor in other studies [11,19,20]. A possible explanation could be that the selection of an adequately high maximum serum basal FSH level limiting chances of clinical pregnancy down to 5% supersedes the effect of age. Additionally, had a larger sample been collected, perhaps age might have become an important predictor of clinical pregnancy and miscarriage. Other studies found clinical pregnancy rates to be about 20% in women under the age of thirty-five years with elevated basal serum FSH levels [21,22]. However, it could also be argued that the FSH cut-off levels used at these specific clinics were not calculated to give a clinical pregnancy rate of under 5%. This therefore represents a difference in the manner of use of the FSH cut-off between this study and those other clinics, in the articles cited.

Analysis of serum FSH showed that only levels above 18 IU/L lead to clinical pregnancy rates to less than 5%. Likely this number changes year to year based on variations in the parameters found to be predictive of clinical pregnancy in this study. From this analysis of serum FSH levels in this population, it can be gathered that FSH-based estimation of IVF clinical pregnancy probabilities likely provides suboptimal information when guiding infertile women. Their personal chances of success with IVF treatment may be greatly underestimated. The findings from this study also suggest that oocyte stimulation, number collected and embryo number are the best predictors for clinical pregnancy. This data would need to be collected during an IVF cycle. Increased FSH levels should not be a reason for women to be turned away from attempting pregnancy with IVF.

As a caveat, we feel it is important to note that while an increased FSH should not limit women from being accepted into an IVF program, the use of a such cut-off should not be disregarded either. While IVF may be a safe procedure with few complications, a physician must still weigh the consequences of those few complications (infection, bleeding, bowel injury, thromboembolism etc.) with the benefits of achieving a live birth [23,24]. Especially in patients with FSH above 18 IU/L in whom we have had minimal success in achieving a clinical pregnancy, let alone a live birth, counselling them against the use of IVF may be more reasonable. These patients should be informed that IVF's high financial, physical and physiological burdens are significant, particularly when there is minimal hope of pregnancy [25]. Therefore, having a set FSH cut-off as part of an overall assessment to allow patients into an IVF program seems acceptable for the above reasons. At the very least, patients should be counselled to their very low likelihood of a live birth.

Strengths of this study include the prospective enrolment of patients and being the first in the literature to perform this type of statistical modeling in this specific cohort. Additionally, an important strength of the study stems from Stanford Reproductive Center not refusing to treat patients using their own gametes based on any abnormal parameters of ovarian reserve and not cancelling cycles for minimal stimulation. Weaknesses of the study include the smaller population size, which may have affected the power to detect some significant predictors of pregnancy. However, it should be noted that several significant predictors were detected with statistical modeling, even at this small sample size, stressing the clinical significance of these predictors.

In summary, increased basal FSH levels should not discourage women from attempting a cycle of IVF. Our findings suggest that it would be beneficial for women with increased FSH levels to attempt a cycle of IVF. Results of ovarian stimulation, especially embryo quantity and quality, appear to be the best predictors of IVF outcomes. Additionally, the serum FSH threshold related to clinical pregnancy rates should be calculated on a yearly basis.
